# Alter egos alter engagement: perspective-taking can improve disclosure quantity and depth to AI chatbots in promoting mental wellbeing

**DOI:** 10.3389/fdgth.2025.1655860

**Published:** 2025-09-10

**Authors:** Christopher You, Rashi Ghosh, Melissa Vilaro, Roshan Venkatakrishnan, Rohith Venkatakrishnan, Andrew Maxim, Xuening Peng, Danish Tamboli, Benjamin Lok

**Affiliations:** ^1^ Department of Computer & Information Science & Engineering, Virtual Experiences Research Group, University of Florida, Gainesville, FL, United States; ^2^ Institute of Food & Agricultural Sciences, University of Florida, Gainesville, FL, United States; ^3^ Department of Computer Science, University of Central Florida, Orlando, FL, United States

**Keywords:** artificial intelligence, chatbot, mental wellbeing, perspective-taking, disclosure, emotional expression, embodied conversational agents

## Abstract

**Introduction:**

Emotionally intelligent AI chatbots are increasingly used to support college students’ mental wellbeing. Yet, adoption remains limited, as users often hesitate to open up due to emotional barriers and vulnerability. Improving chatbot design may reduce some barriers, but users still bear the emotional burden of opening up and overcoming vulnerability. This study explores whether *perspective-taking* can support user disclosure by addressing underlying psychological barriers.

**Methods:**

In this between-subjects study, 96 students engaged in a brief reflective conversation with an embodied AI chatbot. **Perspective-Taking** participants defined and imagined a designated other’s perspective and responded from that viewpoint. **Control** participants provided self-information and responded from their own perspective. Disclosure was measured by quantity (word count) and depth (information, thoughts, and feelings). Additional immediate measures captured readiness, intentions for mental wellbeing, and attitudes toward the chatbot and intervention.

**Results:**

**Perspective-Taking** participants disclosed significantly greater quantity, overall depth, thoughts depth, and frequencies of high disclosures of thoughts and information. Both groups showed significant improvements in readiness and intention to address mental wellbeing, with no difference in improvement magnitude. However, **Control** participants reported significantly lower (better) skepticism towards the intervention and greater increases in willingness to engage with AI chatbots comparatively.

**Discussion:**

This study highlights how perspective-taking and distancing may facilitate greater disclosure to AI chatbots supporting mental wellbeing. We explore the nature of these disclosures and how perspective-taking may drive readiness and enrich the substance of disclosures. These findings suggest a way for chatbots to evoke deeper reflection and effective support while potentially reducing the need to share sensitive personal self-information directly with generative AI systems.

## 1 Introduction

Amid the excitement and rigor of college life, many students encounter mental health challenges that can feel overwhelming and isolating. Recent surveys reveal that over 60% of U.S. college students report experiencing at least one mental health-related issue (e.g., stress, anxiety, depressive symptoms) during their education (1). The most recent Healthy Minds dataset (2023–2024) paints an even starker picture: 78% of students currently indicate some level of need with emotional or wellbeing challenges, yet only 54% had ever reached out to professional counseling, with only 36% doing so in the prior year. Although recent trends may reflect more positivity, available early data reported a median delay of *11 years* between the onset of symptoms and initial treatment among a general U.S. population (2). In response to the rising demand and persistent barriers, research has increasingly turned to digital mental wellbeing support through emotionally intelligent AI chatbots. While other telehealth modalities also aim to expand access, such chatbots offer unique advantages by mitigating time, availability, and location barriers through their asynchronous nature. Numerous studies suggest that emotionally intelligent AI chatbots can even provide interim support for depressive symptoms (3–7). At the same time, recent systematic and meta reviews also highlight limited effectiveness and inconsistent results in addressing mental health concerns (8, 9). Hence, another promising application lies in chatbots’ abilities to empower users to proactively manage their wellbeing or seek additional support from professionals (10). This approach is motivated by evidence suggesting that replacing human care with automated systems in therapeutic contexts can leave users feeling discomfort and reluctance in deeper engagements (11–14). Such self-empowering chatbots have been leveraged to support goal-setting (15), adhere to medication goals (16), drive engagement with online therapy (17), promote smoking abstinence (18), or increase efficacy in addressing eating disorders (19, 20). Rapid advancements of large language models also further improve emotionally intelligent AI chatbots’ abilities to overcome obstacles to promote self-wellbeing (21).

Despite promising developments, meaningful engagement with chatbots for wellbeing is far from guaranteed. Recent reports indicate that U.S. college student adoption of chatbot mental health services remains limited, and attitudes are significantly more negative compared to traditional services (22). Lingering reluctance may result in limited self-disclosure, shallow interaction patterns, and reduced ability to provide meaningful support (23–27). Users may also abandon chatbot interactions due to technical issues, a perceived lack of human emotion or empathy, or doubts in the chatbot’s ability to provide meaningful support (13, 28). Achieving meaningful engagement with chatbots often requires users to self-disclose content that might not otherwise be disclosed due to vulnerability or discomfort. If the goal is to help users feel safe enough to share, then we must also address the psychological mechanisms that underlie emotional risk itself. To do so, many approaches aim to enhance chatbots to normalize stigmas (29, 30) or to become more accommodating in their conversations (e.g., more empathetic, human-like, acceptable) (31–37). However, the emotional burden still largely falls on users: users must choose to open up, risk being vulnerable, and overcome deeply personal inhibitions. Additional challenges arise with respect to privacy and ethical concerns in engaging with AI. Uncertainty in data storage, access, and confidentiality may impede disclosures (38). Moreover, ethical concerns around propagating prejudice due to algorithm bias and limited capabilities for responding to crises raise critical questions about the safety, fairness, and reliability of AI-driven mental health interventions (39, 40). Even if such scenarios are “safe,” deeper consideration for the user may be needed to foster disclosure in such environments.

In an effort to mediate user reluctance in disclosing to chatbots, we propose employing *perspective-taking*. Taking another’s perspective allows one to “discern the thoughts, feelings, and motivations of [others]” (41) and offers emotional distance to reflect on distressing experiences (41–44). Given that users often adopt altered identities in digital contexts (45, 46), interactions with chatbots may offer a unique opportunity to explore identity through perspective taking. We conceptualize taken perspectives in relation to the “self” and “other,” where “other” refers to external entities, such as strangers, friends, family, or even hypothetical entities (44, 47). The ability to take perspectives is one formed in early childhood that is considered critical to empathetic capability (48, 49). Empathy is often attributed to the ability to take perspectives (50): we *share* in other people’s emotions (51) and *reconstruct* their mental states for ourselves (52, 53). Though seemingly intuitive, perspective-taking relies on concrete knowledge to ground inferences about others’ actions (54) and is facilitated by greater self–other overlap (55). As a result, less informed perspectives (i.e., distal constructs) can result in *abstractions*, or the employment of general heuristics and social rules to estimate behavior (44, 56). Pertinent examples include abstract syntax in speech (e.g., more adjectives than descriptive verbs) (44, 57), higher-level terminology in descriptions (58–60), or more polite, indirect language (57, 61).

The primary motivation for perspective-taking in this study is its demonstrated impact on user behavior, attitudes, and outcomes. Perspective-taking can lead to improved prosocial behaviors and intentions of change (62–65). Pahl & Bauer found that briefly adopting the perspective of a young woman affected by environmental changes increased participants’ engagement with environmental materials and enhanced their intentions toward environmental action (66). Perspective-taking with outgroups has been shown to reduce aggression, increase empathy, and diminish stereotyping and bias (67–72). Perspective-taking may also lead to improved outcomes for the self (42, 73–75) Boland et al. found that adopting the perspectives of past selves or others, by receiving or offering compassion, can reduce emotional discomfort and enhance self-compassion (42). Perspective-taking is shaped by factors like altruistic concern and egotistic motivation (76, 77), yet a compelling effect may stem from the merging of self and other (55, 74, 78, 79). Aron and Aron describe this as incorporating others into the self, ultimately viewing them as extensions of oneself (80). Perspective-takers are thought to internalize the insights, thoughts, and emotions of others (81, 82). Perspective-takers may project their own traits onto others (e.g., “I liked this movie; therefore, my friend will too”) (44, 63). They may also adopt traits of others, as seen when taking a professor’s perspective increased self-ratings of intelligence (83). These effects intensify when individuals internalize others’ experiences, emotions, and attributes as part of their self-concept (78, 84, 85).

Similar to prior literature (64, 66, 73), this study investigates perspective-taking as a means to promote behavioral engagement, rather than investigating underlying mechanisms of overlap or attitudes towards the taken other. Specifically, This study examines how perspective-taking can enhance disclosure in conversations with emotionally intelligent AI chatbots. Our utilization of an emotionally intelligent AI chatbot entails an embodied conversational agent (ECA)-guided reflective conversation for addressing ambivalence to change, where theory-driven, AI-generated empathetic expressions of dialogue are adapted and delivered based on individual user disclosures (see Section 2.2.2). Although current approaches have demonstrated that AI systems can detect and express emotion with potential for higher sophistication (33, 86–89), the present study’s integration of an emotionally intelligent AI conversation serves as a *platform* to empirically evaluate perspective-taking. The findings of this work arise from a between-participants study that recruited primarily STEM students from the University of Florida who were randomized into **Perspective-Taking** and **Control** conditions. **Perspective-Taking** participants took the perspective of a self-defined, known other and engaged in the AI-guided conversation fully from the other’s perspective; **Control** participants completed identical tasks with a self-framing and engaged fully from their self-perspective. This study investigated perspective-taking’s effects on the following measures: disclosure (word *quantity* and categorical *depth*), readiness to address mental wellbeing, and attitudes toward the intervention and chatbot. Our main hypotheses predicted **Perspective-Taking** would enhance engagement in the forms of greater disclosure quantities and depths in comparison to the **Control**:

${H1}_{\text{DisclosureQuantity}}$: **Perspective-Taking** participants will exhibit greater quantities of disclosure compared to **Control** participants.

${H2}_{\text{DisclosureDepth}}$: **Perspective-Taking** participants will exhibit greater depths of disclosure compared to **Control** participants.

Secondary to disclosure, we investigated how taking an other’s perspective impacted readiness and attitudes. We hypothesized **Perspective-Taking** participants’ readiness to address mental wellbeing would significantly improve overall from Pre- to Post-measure.

${H3}_{\text{ReadinessOverall}}$: **Perspective-Taking** participants will exhibit improved readiness after the reflective conversation on wellbeing.

Though it was not fully expected that perspective-taking would outperform a self-perspective in a conversation dictated by disclosure and self-reflection, we deferred the remaining outcome hypothesis in favor of the **Perspective-Taking** (experimental) condition:

${H4}_{\text{ReadinessComparison}}$: **Perspective-Taking** participants will exhibit greater improvements on readiness compared to **Control** participants.

${H5}_{\text{AttitudesIntervention}}$: **Perspective-Taking** participants will exhibit more positive attitudes towards the present wellbeing intervention compared to **Control** participants.

${H6}_{\text{AttitudesChatbots}}$: **Perspective-Taking** participants will exhibit greater improvements on attitudes towards AI wellbeing chatbots compared to **Control** participants.

## 2 Materials and methods

Two main conditions were examined to assess the impact of perspective-taking: **Perspective-Taking** (perspective of an other) and **Control** (perspective of self). All participants completed two main intervention steps consisting of perspective-taking tasks and a reflective conversation. The primary difference is in the framing of the tasks themselves (see Table 1).

**Table 1:** Summary of the study conditions, their perspectives, and task framing. Both conditions completed the same steps; however, the framing of the tasks differed based on whether participants took an other’s perspective (**Perspective-Taking**) or engaged as the self (**Control**). A high-level overview of the framing is provided, but see Section 2.1 for specific details.

Condition	Perspective	Task framing
Perspective-Taking	Other	***Imagine** someone in your life who would benefit from discussing their mental health through an online intervention. **Step into their perspective**, consider their experiences, and engage in the tasks ahead… To start, type: “I am ready to play the role of [alias].*
Control	Self	*Engage in this **self-intake** by reflecting on your own experiences and reasons for discussing your mental wellbeing. **Consider your perspective**, experiences, and engage in the tasks ahead… To start, type: “I am ready to begin the conversation.”*

### 2.1 Study design

Prior to the study start, participants selected between a male and female ECA for the remainder of the interaction. The ECA introduced itself and provided an overview of the study based on the participant’s assigned condition. The study consisted of two main phases of interaction described in this section: *perspective-taking* and *reflective conversation*.

#### 2.1.1 Perspective-taking phase

Participants in the **Perspective-Taking** condition were instructed to identify, describe, and imagine another’s perspective during this phase. Rather than being given a fictional persona, participants chose a real person in their life, such as a friend or family member, who would benefit from the reflective conversation. The decision to have participants define a real, known other who might benefit from the interaction was based on prior research and considerations specific to this study. First, perspective-taking may fail due to insufficient information or a lack of reason to set aside egocentric bias and take perspectives (90, 91), and greater proximity will increase the likelihood of adopting an other’s perspective (76). Allowing participants to define a known other who might benefit from the conversation offers greater familiarity with the other’s mental wellbeing and a meaningful reason to take their perspective. Second, establishing conversational depth was a high priority for the study’s wellbeing aims. An other who is too distant from the participant may be difficult to portray, leading to more abstract, higher-level responses (44). Given strong evidence that people can spontaneously take perspectives or empathize without prompting (92–95), allowing participants to define their own target seemed suitable for supporting perspective-taking in this context.

To support effective perspective-taking, the task extended beyond the typical narrative and imaginative phases commonly used in prior studies (50, 66, 96). This study used a persona-crafting task called empathy mapping, which aligns with established perspective-taking processes (54). Given empathy’s strong connection to perspective-taking (62, 92, 97), empathy mapping was appropriate, as it helps participants understand others by viewing the world through their eyes and evokes empathy through persona design (98). Empathy mapping aimed to personify the other by capturing demographic information, personality traits, values, wellbeing concerns, goals, and brief imaginative descriptions of the other’s life. These fields were further informed based on literature in empathy mapping and design of patient personas in eHealth interventions (99–101).

The AI chatbot guided participants through the empathy mapping tasks, explaining the aims and requirements for each prompted item. After designing the persona, participants reviewed their other’s persona and imagined their other’s perspective and experiences. To deepen perspective-taking and ensure participants responded only from their other’s viewpoint, the AI chatbot provided mock conversation prompts for first-person replies as the other. Afterwards, participants began the reflective conversation by entering the following phrase: “I am ready to play the role of [alias]” (where “alias” refers to the other’s defined name). In the **Control** condition, the same empathy mapping and mock scenario tasks were delivered by the AI chatbot to maintain structural parity. The difference in conditions arises in the framing of the task as a personal intake in the **Control**, rather than perspective-taking.

#### 2.1.2 Reflective conversation phase

The reflective conversation was designed to evoke disclosure from participants conducive to building their readiness to address their wellbeing. Therefore, the conversation was designed using principles from motivational interviewing, a client-centered approach to enhance readiness, resolve ambivalence, and encourage capability and autonomy (102, 103). To enable meaningful comparison of disclosure quantity and depth, a fixed set of conversation items replaced the open-ended format typical of motivational interviews. This design allowed participants to receive the same, verbatim open-ended questions across interactions. Furthermore, the AI chatbot was designed to convey empathy and emotion in response, which can influence disclosure attitudes (104, 105). A structured conversation also afforded consistency in the quantity and depth of empathetic expression from the AI chatbot. The rule-based reflective conversation script was designed by two authors MV, an expert in health communication trained in motivational interviewing, and CY, who received the standard full-day training in motivational interviewing (106). A formal pilot with (*n* = 58) participants was conducted while iteratively updating the conversation’s script.

Four classifications of motivational interviewing strategies reviewed by Hardcastle et al. served as sub-phases for the present reflective conversation (107). In line with motivational interviewing’s open nature, the authors describe their classifications as identified themes rather than *strict* design requirements for conversations. Their classifications include motivational interviewing strategies for *engaging*, *focusing*, *evoking*, and *planning*. Although the conversation includes both closed- and open-ended items per Hardcastle et al., we focus here on the *nine* open-ended disclosure items (see Section 2.3.1). These nine disclosure items include strategies directly from three of the four classifications: **Engaging**: *two* “open-ended question“ disclosure items, **Evoking**: *five* disclosure items on “troubleshooting” barriers to change, “looking forward” on future possibilities, “identifying past successes” in coping strategies, “exploring values” relating to the wellbeing concern or behavior, and “brainstorming” options to change, and **Planning**: *two* disclosure items of “considering change options” and “developing a change plan” towards one concrete, self-designed next step (see Table 2) (107). In *Focusing*, participants were provided the opportunities to engage in a set of resources (NIMH & CDC) containing techniques for improving mental wellbeing through closed-ended responses.

**Table 2:** The specific strategies employed for the conversation’s nine disclosure items and empathetic expressions. The disclosure strategies refer to the nine open-input items analyzed, and the empathetic expression strategies illustrate how prompts were designed (107).

Conversation phase	Disclosure items	Disclosure strategies	Empathetic expression strategies
Engaging	Q1	Open-ended question	Offer emotional support and reflective statements
	Q2	Open-ended question	Offer emotional support and reframing
Focusing	N/A	N/A	N/A
Evoking	Q3	Troubleshooting	Coming alongside and normalizing
	Q4	Looking forward	Affirmations and emphasize autonomy
	Q5	Identify past successes	Reframing and affirmations
	Q6	Values exploration	Offer emotional support and reflective statements
	Q7	Brainstorming	Reframing and agreement with a twist
Planning	Q8	Considering change options	Permission to provide information and advice and emphasize autonomy
	Q9	Develop a change plan	Support change/persistence and emphasize autonomy

Participants completed the reflective conversation by responding to the AI chatbot’s nine disclosure items and additional closed-ended items across the four sub-phases. With each disclosure response from the participant, the AI chatbot employed a sequence of providing empathetic expression before delivering the ensuing prompt (see Section 2.2.2 for implementation details). To ensure proper study completion, static interface messages reminded participants to engage from the defined perspective, and the AI chatbot’s first question requested participants to provide their defined alias for conversation. Participants who provided an alias differing from their taken perspective were removed from analyses. All participants completed the reflective conversation’s disclosure and closed-ended items across four sub-phases. **Perspective-Taking** participants responded from the designated other’s perspective, while **Control** participants responded from their own. Following the conversation, participants engaged in a short transitional phase to return to their own perspective, labeled self-reflection in the **Control** group. During this transition, participants were told to momentarily pause to reorient to their own perspective and experiences and self-reflect on the conversation. **Perspective-Taking** participants were reminded to complete post-surveys from their own perspective, as in the pre-survey.

### 2.2 AI chatbot and empathetic expression protocol

This section describes the architecture for the AI chatbot and the design for empathetic expression in responses. The AI chatbot interaction is built on a Node.js framework, which is commonly used to build and deploy web applications. The study is deployed asynchronously over the web, where participants were required to complete the study on a desktop or laptop web-enabled device.

#### 2.2.1 AI chatbot

**Figure 1:**
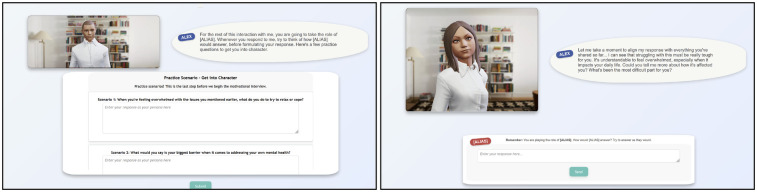
The intervention interfaces illustrating the male and female ECA. (Left) mock conversation scenario in perspective-taking phase and (Right) sample disclosure item Q2 in reflective conversation phase. Avatar created using https://readyplayer.me/.

The generation of ECAs, their verbal responses, and their corresponding non-verbal behaviors are described. ECAs were employed as evidence has indicated they can provide a level of human touch and foster greater willingness to disclose (108–110). Each ECA is designed via ReadyPlayerMe, a free-to-use online tool to generate 3D models that can be rigged, rendered, and utilized on the web using a Three.js library. ReadyPlayerMe provides integrated blendshapes to the model, which support the non-verbal behaviors of animation and lip-syncing. A male and female ECA options were generated (see Figure 1) and included in the pilot tests with the (*n* = 58) participants to broadly check for any negative sentiments in design choices. Upon accessing the study’s webpage, the ReadyPlayerMe-exported ECA model is loaded and rendered on users’ devices.

When a participant interacts with the ECA, verbal responses containing text and audio are generated statically or dynamically using the rule-based conversation script and LLMs. To generate the verbal response, OpenAI’s Completions (4o-mini) and Text-To-Speech (tts-1) models are employed (male voice: *echo*; female voice: *shimmer*). The conversation script indicates how text and audio responses should be *static*ally or *dynamic*ally generated. Static verbal responses are pre-generated to control the interactions so that participants receive identical responses when necessary. Static verbal responses from the ECA include the nine disclosure items in the reflective conversation or the empathy mapping items in the perspective-taking phase. Both conditions followed the same rule-based conversation script during the reflective conversation, producing identical static verbal responses. However, the perspective-taking phase used two distinct scripts due to differences in the empathy mapping framing. In contrast, dynamic verbal responses are generated in real-time based on individual participant queries across both conditions. Dynamic verbal responses largely pertain to the empathetic expressions delivered in the *reflective conversation*. Where the script calls for a dynamic verbal response, an empathetic expression strategy guides the LLM (see Section 2.2.2). The ECA immediately responds with a verbal backchannel (e.g., “Thanks for being open. I’m working on generating something thoughtful based on what you’ve shared.”) to acknowledge input and mask LLM response delays. The system generates dynamic verbal responses and queues them to deliver after the verbal backchannel finishes.

The ECAs perform non-verbal behaviors such as animations and lip-syncing that correspond to their verbal responses, implemented using the open-source repository *TalkingHead*.^1^ For animations, the ECA employs template behaviors when idling or speaking (e.g., standing straight, leaning to the side, gestures). When the ECA is idle, a sequence of randomized idle poses with a generic breathing animation is rendered. When the ECA is speaking (i.e., when a verbal response is delivered), additional animations were integrated alongside the randomized talking poses (e.g., a wave when the ECA introduces itself). For lip-syncing, transcriptions and timestamps of the spoken audio are derived from the verbal responses. The transcription is processed to extract individual phonemes, which are mapped to corresponding visemes. These visemes are coded in the lip-sync system using Oculus Lipsync and *TalkingHead*. Timestamps show when to apply visemes to the ECA’s facial blendshapes during playback to simulate natural speech. In sum, a typical conversation turn will entail: receiving user input, delivering verbal backchannels, generating appropriate (dynamic) verbal response for empathetic expression, retrieving subsequent (static) verbal response for disclosure or closed-ended item, delivering entire verbal response, animating non-verbal behaviors, and synchronizing lip movements to verbal response.

#### 2.2.2 Empathetic expressions of dialogue

While numerous articles explore opportunities to detect and convey emotion accordingly (33, 86–89), the present work primarily focuses on how emotionally intelligent systems can be further enhanced by psychological theories of perspective-taking and self-distancing. However, establishing emotional intelligence from the AI chatbot remains critical to the study’s mental wellbeing design and in understanding how perspective-taking can enhance such chatbots. Thus, we designed the AI chatbot to convey empathetic expression strategically to individual participant disclosures. In health communications, there are *opportunities* (when) empathy must be conveyed and corresponding *expressions* or representations (what) of empathy (111, 112). With AI chatbots, frameworks suggest that similar processes of recognition and communication can be administered (113, 114). Focusing on perspective-taking, this study aims to streamline the process using a rule-based, structured approach with LLMs. The rules determine *when* to prompt during the nine open-ended disclosure items, while the LLMs use motivational interviewing strategies to generate *what* to say through empathetic expressions.

The AI chatbot’s empathetic expressions are based on Hardcastle et al.’s motivational interviewing strategy classifications, which also guided the design of the disclosure items (107). The present study’s empathetic expression strategies are primarily derived from relational strategies within the four classifications. The nature of the previous disclosure item primarily guides the empathetic expression used in the *reflective conversation* script. Open-ended questions like Q1 and Q2 engage participants by asking them to describe a mental wellbeing concern or goal and its impact on them. Hardcastle et al.’s strategies of offering emotional support, summarization/reflective statements, and reframing help participants feel heard and encourage reflection. In questions that help the participant *plan*, like in Q8 and Q9, it is more important to emphasize autonomy in the participant’s choice and support their change and persistence (102). Table 2 illustrates the specific disclosure and empathetic expression strategies utilized in the present study, as listed directly from Hardcastle et al.’s classifications (107). For each disclosure, emotional dialogue expressions in dynamic verbal responses are generated by identifying the relevant theory-driven strategy, adapting to user disclosures and conversation history, and prompting the AI model accordingly. By anchoring each empathetic expression in an established theoretical classification, the present study provides a controlled and interpretable environment to assess the impact of perspective-taking in conversations within AI chatbots that are intelligent to user disclosures with their expressions of emotion.

### 2.3 Measures

To address the hypotheses, three primary constructs were investigated: disclosure, readiness, and attitudes.

#### 2.3.1 Disclosure

Based on prior literature (115, 116), disclosure is assessed through measures of **quantity** and **depth**. For ${H1}_{\text{DisclosureQuantity}}$, LIWC-22 was used to capture *word counts* across the nine disclosure items to determine if the disclosed quantity of words was altered by the perspective-taking manipulation (117). To supplement analyses on disclosure quantity, we calculated an *abstractness* score (1–5) across participants’ nine disclosure responses, using the Linguistic Category Model (LCM) (118, 119). Seih et al.’s generated LIWC-22 dictionary and their described process for using the *TreeTagger*^2^ tool was used to capture frequencies for LCM (120, 121).

For ${H2}_{\text{DisclosureDepth}}$, qualitative analysis was performed using the process and categories defined by Barak & Gluck-Ofri to code each of the nine participant responses in terms of *information*, *thoughts*, and *feelings* (122). Each response was segmented into distinct statements and categorized as follows: *information*, when the writer shared personal details, experiences, or factual content; *thoughts*, when they expressed personal opinions or reflections; and *feelings*, when they conveyed emotional or affective responses. Within each category, one of three levels of depth is assigned: 1. no disclosure about the user in the category altogether, 2. a disclosure about the user but in general or mild expressions, 3. a disclosure about the user in personally revealing, intimate, or deep expressions. Therefore, each response will have resulted in a score (1–3) for all categories of information, thoughts, and feelings. An *overall depth* score for the amount of disclosure is obtained by combining the levels of information, thoughts, and feelings for each response (122). Sample responses categorized as depth levels 1, 2, and 3 for each category can be found in Table 3.

**Table 3:** Sample responses illustrating coded depths (1–3) of information, thoughts, and feelings from our study population. Each statement will always receive three codes; therefore, statements shown in this table may have received different scores for their non-represented categories (e.g., [P46] was rated as (depth = 1) no disclosure of feelings and (depth = 3) high disclosure of information).

Depth	Information	Thoughts	Feelings
1. No disclosure	***[P33]** My girlfriend is the best*	***[P44]** Projects take a long time to complete*	***[P46]** I have used breathing techniques and meditation. also talking to people and not isolating myself [has worked so far].*
2. Low disclosure	***[P38]** Just letting my thoughts roam [has not been] effective sometimes as it leads me to start off on one thing and by the end I will have thought through 20 different things and I forget how I even got there.*	***[P40]** My friends [inspire me to take action]. I want to be the best version of myself for them to be a good friend.*	***[P9]** Today, I am feeling okay*… *I don’t feel as bad.*
3. High disclosure	***[P47]** I tried therapy, but I didn’t like it so I stopped. I refuse to take medication to try and help my mental health. So far, nothing I’ve done has worked for me so far.*	***[P11]** I remember when he was young and loved spending time with me and talking to me, and now it just seems like he hates me. I wish we could be close like before!*	***[P3]** When I’m alone, I often feel these dark thoughts. I’m very angry, sad, and confused about the whole situation*

A total of 96 participants properly completed the entire intervention, but disclosure analysis includes 55 participants’ responses to the disclosure items: **Control** (*n* = 29 participants × 9 items = 261 items) and **Perspective-Taking** (*n* = 26 participants × 9 items = 234 items). Technical errors early in data logging prevented the capture of conversation logs for the outstanding participants. The resulting disclosure analysis includes a robust set of (*n* = 493 items × 3 codes = **1479**) codes, after validating responses and omitting (*n* = 2) responses due to invalid input. Authors AM, DT, and XP served as three independent coders with condition- and participant-anonymized, shuffled versions of the conversation transcripts. Each author had prior experience in qualitative methods and received training on the process by Barak & Gluck-Ofri before individually coding the same 30% subset of the data (*n* = 615 codes). Kendall’s W indicated the three coders statistically significantly agreed in their assessments, W=.856,p<.001. Disputes within responses were settled as a group, and each coder individually coded a third of the remaining data. See Table 4 for quantities of depth at each level across participants’ nine disclosure items.

**Table 4:** Table illustrates the frequencies (n and %) of depth codes for each condition in terms of the categories of *Information*, *Thoughts*, and *Feelings*.

Depth of disclosure	Control						Perspective-Taking					
	*Information*		*Thoughts*		*Feelings*		*Information*		*Thoughts*		*Feelings*	
	n	%	n	%	n	%	n	%	n	%	n	%
1. No disclosure	110	42.3	135	**51.9^a^**	246	94.6	108	46.4	95	**40.8^a^**	226	97.0
2. Low disclosure	83	**31.9^b^**	66	25.4	6	2.3	45	**19.3^b^**	50	21.4	1	0.4
3. High disclosure	67	**25.8^c^**	59	**22.7^d^**	8	3.1	80	**34.3^c^**	88	**37.8^d^**	6	2.6
**Total Counts**	260	100	260	100	260	100	233	100	233	100	233	100

Significantly different proportions are illustrated by matching **bold superscripts: ^a, b, c, d^** (e.g., superscript ^**d**^ refers to a significantly greater proportion of high disclosure (depth = 3) of *Thoughts* in **Perspective-Taking** compared to **Control**).

#### 2.3.2 Readiness

To assess readiness for wellbeing change, readiness is assessed through measures of **stage** of readiness, **composite** readiness score, and **intentions** to address mental wellbeing. For stage and composite, we collected responses to the *Readiness-to-Change Questionnaire* (123). This questionnaire is grounded in the Transtheoretical Model of Change (TTM) (124), a structured and theoretical framework commonly used in health interventions and digital health (125, 126) to conceptualize behavior change as a progression through distinct stages (127). Computational modeling of TTM has demonstrated its validity in classifying users into these stages (128), and TTM-based digital interventions have shown efficacy in promoting behavioral change (129). When combined with empathetic communication strategies in chatbots, TTM-based assessments can enhance responsiveness to users’ psychological needs (113). We assess an individual’s readiness to change *stage* across three stages: **P**re-**C**ontemplation, **C**ontemplation, and **A**ction (130). The stage measure indicates whether the person is not yet considering change (PC), thinking about making a change (C), or actively working toward change (A). The *composite* readiness measure is produced by the following equation: C+A−PC. Additionally, one single item was adapted from prior work to assess participant *intention* to address their mental wellbeing, Pre and Post (131, 132). ${H3}_{\text{ReadinessOverall}}$ investigates within-condition changes from Pre to Post for the measures of stage, composite readiness, and intent. ${H4}_{\text{ReadinessComparison}}$ investigates between-condition changes from Pre to Post for the measures of stage, composite readiness, and intent.

#### 2.3.3 Attitudes

The attitudinal metrics include a questionnaire on participant attitudes towards the present study’s wellbeing chatbot intervention (**skepticism, confidence, technologization threat, anonymity**) and a single-item measure on **willingness** to engage with AI *chatbots* for mental wellbeing. For ${H5}_{\text{AttitudesIntervention}}$, attitudes were measured through an adaptation of the Attitude towards Psychological Online Interventions (APOI) Questionnaire (133). The scale comprises four dimensions: *skepticism* and perception of risks, *confidence* in effectiveness, *technologization* threat, and *anonymity* benefits. For ${H6}_{\text{AttitudesChatbots}}$, attitudes of willingness to engage with AI chatbots was measured Pre and Post through a single item similar to the prior intention metric.

### 2.4 Procedure

We conducted a between-participant study with the described system with undergraduates at the University of Florida. Participants selected a time to participate in the study through one of the university’s research recruitment platforms, which provides course credit to students as compensation for research studies. After giving informed consent, participants completed the pre-survey measures of readiness listed in Section 2.3. Participants were then randomized into one of **Perspective-Taking** or **Control**. Each participant completed the intervention steps of *perspective-taking* and *reflective conversation* as described in Section 2.1. Concluding the intervention steps and reflection in their self-perspective, participants completed the post-survey measures of readiness and attitudes described in Section 2.3, as well as demographics. Participants were debriefed on the study concerning how their anonymized data would be used and were subsequently granted course credit for their participation.

### 2.5 Participants

An a priori power analysis using G*Power was conducted for a mixed-design ANOVA with 2 groups (between-subjects factor) and 2 time points (within-subjects factor). Assuming an α = 0.05, power (1−β)=0.95, a medium effect size of f = 0.20, a correlation among repeated measures = 0.5, and sphericity met, we yield the minimum accepted sample size of N≥84. This study was approved by the University of Florida Institutional Review Board, and all participants provided written informed consent. To account for dropout and errors in completion, a total of 99 participants were recruited via the research recruitment platform and completed the entirety of the Procedure in Section 2.4. Three (*n* = 3) **Perspective-Taking** participants were excluded from analyses for introducing themselves as an alias deviant from their defined perspective’s alias (see Section 2.1.2). The final analysis included 96 participants, with (*n* = 48) participants each in the **Control** and **Perspective-Taking** conditions.

Participants ranged in age from 18 to 41 years (M=21.8,SD=2.98). Gender identities included 67% male, 29% female, and 4% non-binary or unreported. Demographics were 52% White, 36% Asian or Pacific Islander, 4% Black or African American, 4% mixed, and 4% unreported, with 16% also identifying as Hispanic or Latino. In terms of education, all participants were students at the University of Florida, with 79% attending as undergraduate students and the remainder as graduate students. As the employed recruitment platform provides compensation for computer science-related courses, breakdowns of majors largely pertain to STEM: 71% computer science-related, 22% engineering, 5% mathematics or education-related, and 2% unlisted.

## 3 Results

After collection and coding, data pre-processing was conducted using Python (3.12.2). Statistical analyses were primarily performed using IBM SPSS Statistics (Version 30). Descriptive statistics were computed to summarize the key variables across conditions. The significance level was set at p<0.05, and assumptions for each test (e.g., normality tests via Shapiro-Wilk) were evaluated before conducting the analyses. Assumptions for independent samples *t*-tests and ANCOVAs, using Pre as a covariate, were tested and revealed violations of normality in measures, p<0.05. Therefore, Mann-Whitney *U* tests and aligned rank transform (ART) ANOVAs, with Condition (Control and Perspective-Taking) and Time (Pre and Post) as factors, were conducted for each measure. Effect sizes were calculated and listed via rank-biserial correlation (r), partial eta squared $\eta_{p}^{2}$, and Cramer’s V for the corresponding non-parametric tests and Chi-square tests. Post hoc pairwise comparisons for ART ANOVAs were performed using ART-C with a Holm correction across the six pairwise post hoc comparisons to control the familywise error rate (134, 135). Due to a lack of support for ART ANOVAs in SPSS (see Section 3.2 for analysis), ART ANOVAs were analyzed in R (4.5.0) using ARTool (135).

### 3.1 Disclosure

#### 3.1.1 Quantity

**Figure 2:**
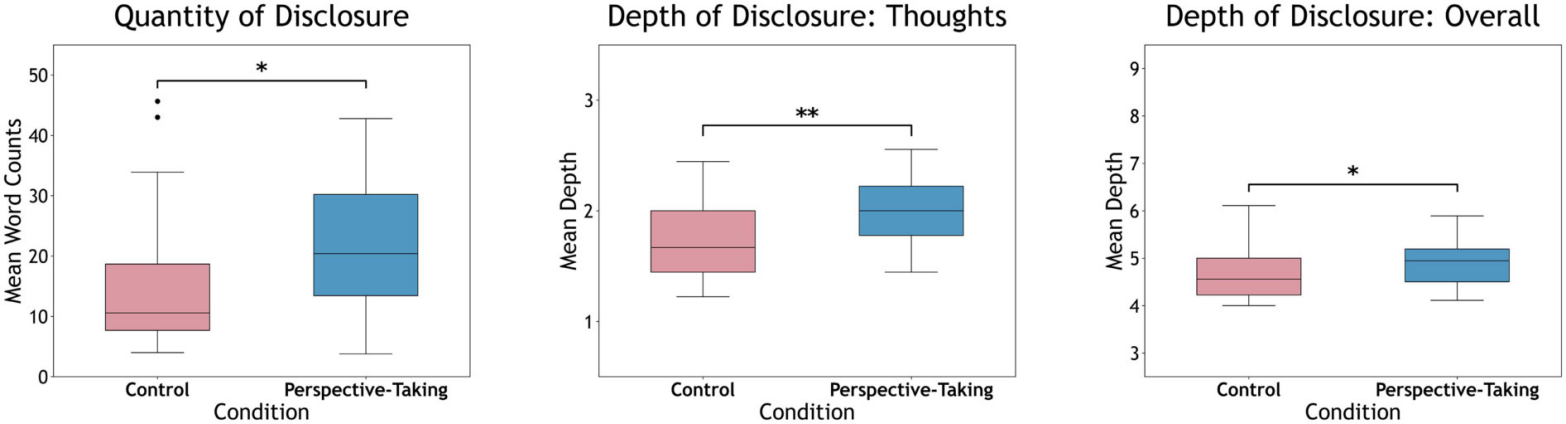
Box plots with medians for disclosure for **Control** and **Perspective-Taking** in terms of (Left) quantities, (Middle) depth of thoughts, and (Right) depth overall. Quantity, depth of thoughts, and depth overall refer to means for word counts, depth (intimacy) of thoughts, and depth (intimacy) of overall content, respectively, across the nine disclosure items, with significance illustrated (**<0.01, *<0.05).

**Word Count**. Mann-Whitney *U* test found a significant difference between conditions in word counts (averaged across the nine disclosure items), U=523,z=2.45,p=0.014,r=0.33. Word counts were significantly higher in **Perspective-Taking** (Mdn = 20.2, M = 21.9, SD = 11.7) compared to **Control** (Mdn = 10.4, M = 14.7, SD = 10.3) (see Figure 2).

**Abstractness**. Mann-Whitney *U* test revealed no significant difference in abstractness via LCM scores between conditions, U=380,z=0.051,p=0.960. Descriptives for abstractness (1 = concrete, 5 = abstract) are included for reference: **Perspective-Taking** (Mdn = 3.22, M = 3.27, SD = 0.243) and **Control** (Mdn = 3.22, M = 3.24, SD = 0.179).

#### 3.1.2 Depth

In addition to Mann-Whitney *U* tests, Chi-square tests for homogeneity were employed to assess frequencies of depth 1, 2, or 3 for each category of information, thoughts, and feelings. Post hoc pairwise comparisons for Chi-squares were conducted using *z*-tests with a Bonferroni correction.

**Information**. Mann-Whitney *U* test revealed no significant difference in depth of information disclosure between conditions, U=433,z=0.952,p=0.341.

Chi-square and post hoc tests indicated a significantly greater proportion of high disclosures (depth = 3) for **Perspective-Taking** compared to **Control**, $\chi^{2}(2)=11.0,p<0.01,V=0.15$. In turn, a significantly lower proportion of low disclosures (depth = 2) was found for **Perspective-Taking** compared to **Control**. See Table 4 for coded depth frequencies and differences in Information.

**Thoughts**. Mann-Whitney *U* test found a significant difference in depth of thoughts disclosure between conditions, U=549,z=2.92,p<0.01,r=0.39. Depth of thoughts were significantly higher in **Perspective-Taking** (Mdn = 2.00, M = 1.97, SD = 0.318) compared to **Control** (Mdn = 1.67, M = 1.71, SD = 0.320) (see Figure 2).

Chi-square and post hoc tests indicated a significantly greater proportion of high disclosures (depth = 3) for **Perspective-Taking** compared to **Control**, $\chi^{2}(2)=13.4,p<0.001,V=0.16$. In turn, a significantly lower proportion of no disclosures (depth = 1) was found for **Perspective-Taking** compared to **Control**. See Table 4 for coded depth frequencies and differences in Thoughts.

**Feelings**. Mann-Whitney *U* test revealed no significant difference in depth of feelings disclosure between conditions, U=333,z=−0.938,p=0.348.

There was a heavy skew in scores with no disclosure of feelings (depth = 1). Fisher’s exact test was conducted due to an inadequate sample size for the chi-square test of homogeneity (136). The distributions of feelings depth scores were not significantly different between conditions, p=0.224.

**Overall Depth**. Mann-Whitney *U* test found a significant difference in overall disclosure depth between conditions, U=504,z=2.15,p=0.032,r=0.29. Overall depth was significantly higher in **Perspective-Taking** (Mdn = 4.94, M = 4.90, SD = 0.519) compared to **Control** (Mdn = 4.56, M = 4.63, SD = 0.530) (see Figure 2).

### 3.2 Readiness

**Figure 3:**
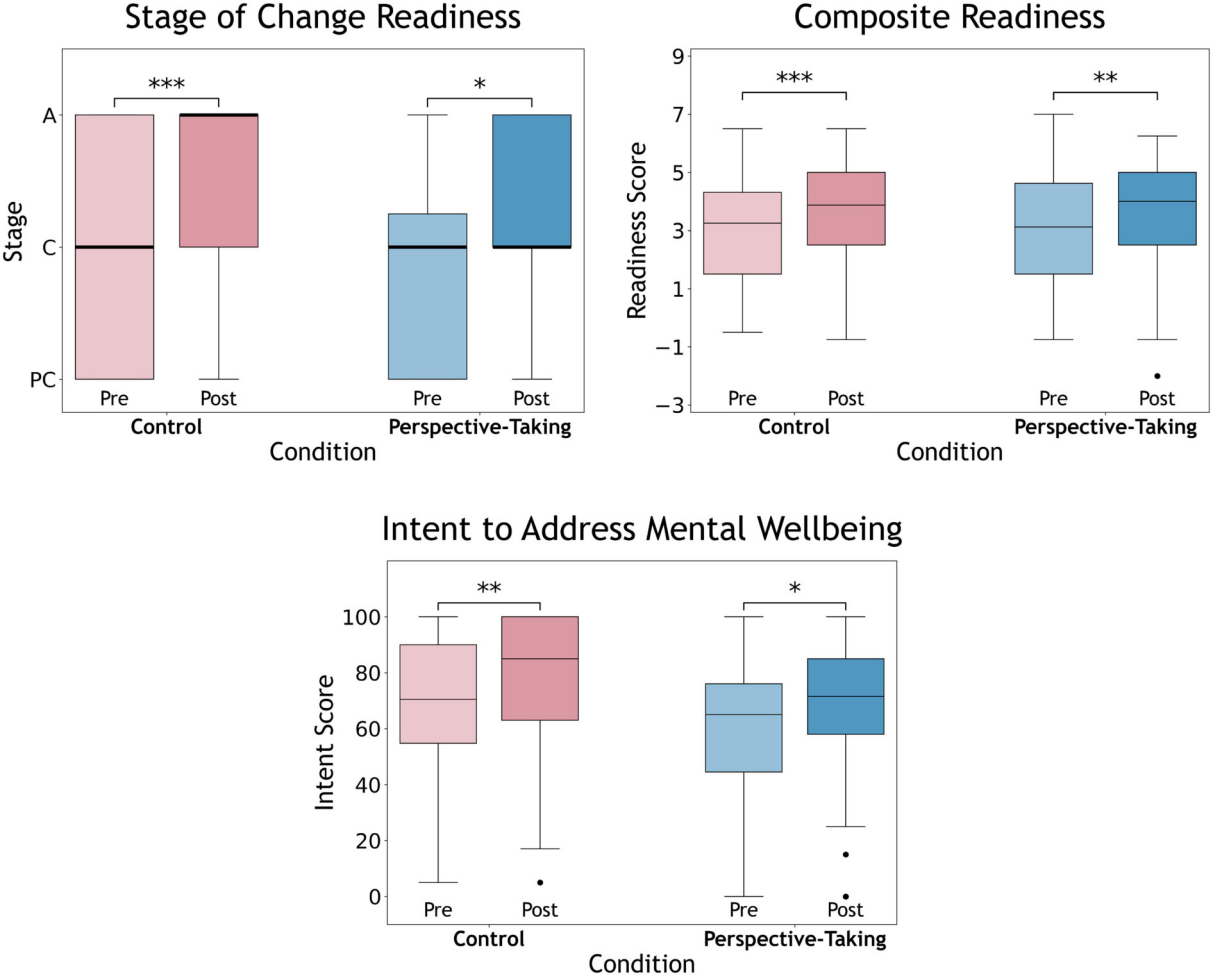
Box plots of Pre- and Post-readiness measures for **Perspective-Taking** and **Control** with medians: (Top-Left) stage of readiness for **P**re-**C**ontemplation, **C**ontemplation, and **A**ction, (Top-Right) composite readiness scores, and (Bottom) intent to address wellbeing. Significance within conditions from Pre to Post illustrated (***<0.001, **<0.01, *<0.05). No significant effects of Condition × Time interaction.

**Stage**. The ART ANOVA revealed a significant main effect of Time, $F(1,94)=17.5,p<0.001,\eta_{p}^{2}=0.157$, indicating an overall improvement in stage of readiness from Pre to Post across conditions. Post hoc analyses revealed a significant improvement in stage from Pre to Post for **Control** only (t(94)=3.58,p<0.01,r=0.35). No significant main effect of Condition or Condition × Time interaction was found, suggesting that the magnitudes of improvement over time did not differ significantly. Separate analysis on the deltas from Pre-stage to Post-stage also found no significant difference between conditions, p>0.05 (see Figure 3).

**Composite**. Similar to *Stage*, the ART ANOVA revealed a significant main effect of Time, F(1,94)=27.6,p<0.001, $\eta_{p}^{2}=0.227$, indicating an overall improvement in composite readiness scores from Pre to Post across conditions. Post hoc analyses revealed significant increases in composite readiness from Pre to Post for each condition: **Perspective-Taking** (t(94)=3.45,p<0.01,r=0.34) and **Control** (t(94)=4.06,p<0.001,r=0.39). No significant main effect of Condition or Condition × Time interaction was found, suggesting that the magnitudes of improvement over time did not differ significantly. Separate analysis on the deltas from Pre-composite readiness to Post-composite readiness also found no significant difference between conditions, p>0.05 (see Figure 3).

**Intention**. ART ANOVA revealed a significant main effect of Condition, $F(1,93)=6.43,p=0.013,\eta_{p}^{2}=0.065$. Post hoc comparisons for Condition revealed significantly higher overall intentions in the **Control** compared to **Perspective-Taking**, p=0.013,r=0.25. A significant main effect of Time was also observed, $F(1,93)=18.9,p<0.001,\eta_{p}^{2}=0.169$, indicating an overall improvement in intention to address mental wellbeing from Pre to Post across conditions. Post hoc analyses revealed significant increases in intentions from Pre to Post for each condition: **Perspective-Taking** (t(93)=2.85,p=0.022,r=0.28) and **Control** (t(93)=3.48,p<0.01,r=0.34). No significant effect of Condition × Time interaction was found, suggesting that the magnitudes of improvement over time did not differ significantly. Separate analysis on the deltas from Pre-intention to Post-intention also found no significant difference between conditions, p>0.05 (see Figure 3).

### 3.3 Attitudes

**Figure 4:**
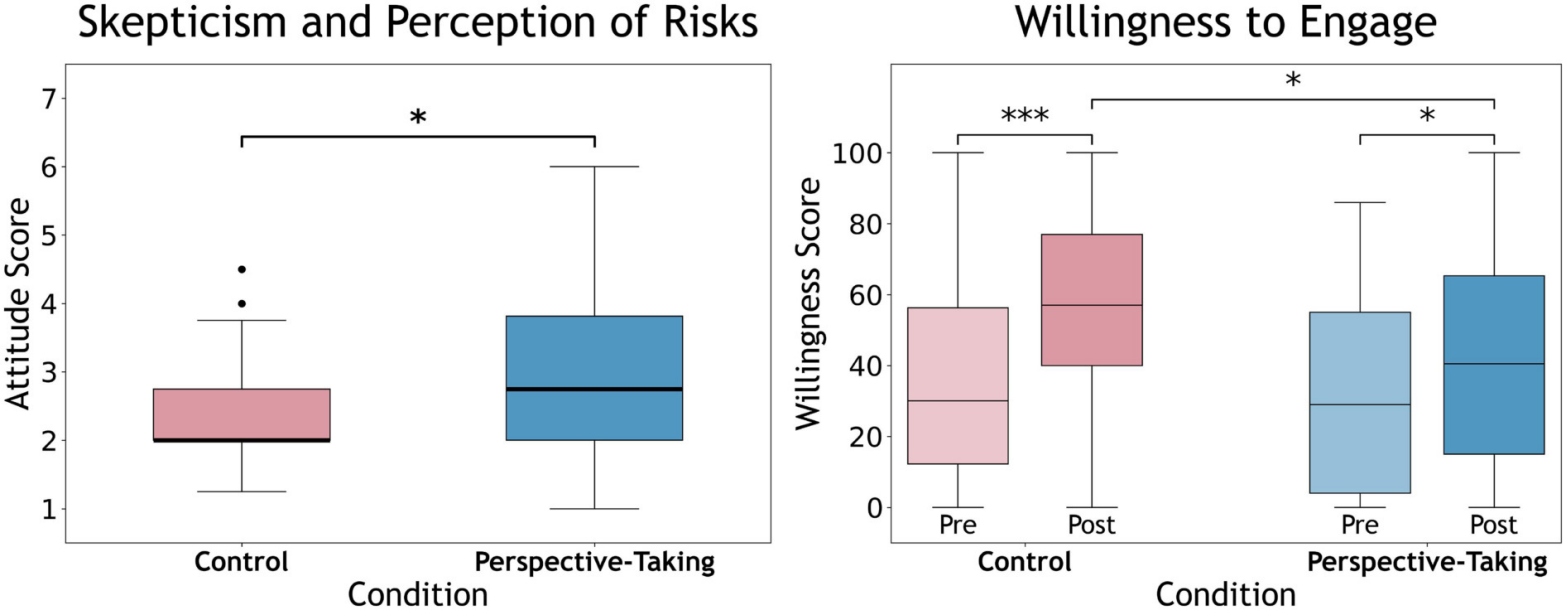
Box plots of attitudinal measures for **Perspective-Taking** and **Control** with medians: (Left) skepticism and perception of risks, and (Right) Pre- and Post-willingness to engage with AI chatbots for mental wellbeing. Significance in box plot between conditions and Pre to Post differences illustrated (***<0.001, *<0.05). There was a significant interaction effect of Condition × Time in willingness to engage in favor of **Control**.

**Skepticism and perception of risks**. Mann-Whitney *U* test found a significant difference in skepticism and perception of risks between conditions, U=1558,z=3.02,p<0.01,r=0.31. Skepticism and perception of risks were significantly higher (worse) in **Perspective-Taking** (Mdn = 2.75, M = 3.09, SD = 1.33) compared to **Control** (Mdn = 2.00, M = 2.31, SD = 0.733) (see Figure 4).

**Confidence in effectiveness**. Mann-Whitney *U* test revealed no significant difference in confidence in effectiveness between conditions, U=961,z=−1.42,p=0.155.

**Technologization threat**. Mann-Whitney *U* test revealed no significant difference in technologization threat between conditions, U=1122,z=−.221,p=0.825.

**Anonymity benefits**. Mann-Whitney *U* test revealed no significant difference in anonymity benefits between conditions, U=1161,z=0.063,p=0.950.

**Willingness to engage.** ART ANOVA revealed a significant main effect of Time, $F(1,93)=45.9,p<0.001,\eta_{p}^{2}=0.331$, indicating an overall improvement in willingness to engage with AI chatbots for mental wellbeing from Pre to Post across conditions. Post hoc analyses revealed significant increases in willingness from Pre to Post for each condition: **Perspective-Taking** (t(93)=3.09,p=0.010,r=0.31) and **Control** (t(93)=5.72,p<0.001,r=0.51). A significant Condition × Time interaction was also observed, F(1,93)=5.23,p=0.024, $\eta_{p}^{2}=0.053$, suggesting that the effect of time differed between conditions. Additionally, the **Control** reported significantly greater willingness to engage with AI wellbeing chatbots at Post compared to **Perspective-Taking**, p=.032,r=0.22 (see Figure 4).

## 4 Discussion

Our results suggest that perspective-taking can significantly alter the ways in which users disclose to chatbots. In line with hypotheses ${H1}_{\text{DisclosureQuantity}}$ and ${H2}_{\text{DisclosureDepth}}$,textbfPerspective-Taking participants disclosed significantly greater word quantities, depth of thoughts, and overall depth than **Control** participants. Perspective-taking also resulted in more frequent high-depth (level 3) disclosures in both information and thoughts compared to the control. Results also showed significant improvement in all readiness measures across both conditions, supporting our hypothesis ${H3}_{\text{ReadinessOverall}}$. Improvements in readiness did not support our deferred choice of **Perspective-Taking** for ${H4}_{\text{ReadinessComparison}}$, but surprisingly, we also found no interaction between Condition and Time, nor any difference in the rate of change (deltas) across readiness measures. The promising effects are tempered by attitudes: **Perspective-Taking** participants showed significantly greater skepticism and a less pronounced increase in willingness to engage with wellbeing chatbots than **Control** participants, contrary to ${H5}_{\text{AttitudesIntervention}}$ and ${H6}_{\text{AttitudesChatbot}}$. We interpret the findings observed on disclosure and the implications of this work for wellbeing chatbots accordingly.

### 4.1 Interpreting effects on disclosure

To contextualize the effects of perspective-taking on disclosure, we provide interpretations of the improved disclosure, consider the nature of the disclosures, and identify limits to our disclosure findings.

Perspective-taking significantly improved the quantity and depth of participants’ disclosures (see Figure 2). Our findings echo prior work showing that perspective-taking can shift engagement behavior in applied contexts, now extended to chatbot-mediated disclosures (66, 137, 138). Such literature suggests perspective-takers often align their behavior with their expectations of the other’s imagined actions, which can override intrinsic behavioral constraints (50, 54, 62, 76, 91). In the present study, we suggest that the change in disclosure behavior stems from similar effects and indicates greater substance within these disclosures, rather than an abstract increase in verbosity. Despite prior claims that distal constructs and psychological distance promotes abstraction (44, 139), we observed no such increase in abstract language among perspective-taking disclosures. The observed improvement was also not limited to quantity, as the depth of thoughts and overall disclosures were significantly greater when perspective-taking. This would indicate that **Perspective-Taking** participants illustrate a greater disclosure of personal and intimate information (115, 140). While quantity and depth can often relate, lower quantity disclosures can still result in higher depths (122), and their correlations are not necessarily positive (141). The observed findings of improved disclosure depths further suggest that perspective-taking fostered greater substantive content from users, rather than a simple inflation of abstract or verbose wording.

While disclosure quantity and depth seem to have meaningfully improved, it is worthwhile to discuss the nature of the disclosures produced in the **Perspective-Taking** condition. A natural question that arises is in the self-relevance of these disclosures, as they were uttered wholly from the perspective of the designated other. Even if the disclosures do not directly mirror personal information or thoughts, prior literature notes that individuals often project self-relevant traits onto imagined others (44, 56). Although the present study did not directly measure overlap, the study design of self-designation for an other, imagined in the first person, aimed to promote overlap and afford successful perspective-taking (54, 142). Our earlier discussion on a lack of abstractness differences would also align with such literature (56, 90, 139). Even further, the results also demonstrate that **Perspective-Taking** participants’ readiness improved, despite disclosing from an other’s perspective. The readiness gains may reflect previously documented merging effects (55, 79, 80). Together, the study design and findings suggest plausibility that **Perspective-Taking** participants projected some properties of the self (albeit, to a likely lesser extent than **Control**) in their estimations of the other, resulting in the improved readiness outcomes. We ultimately still characterize our findings as an improvement in *disclosure*, rather than *self*-disclosure, recognizing the limitations in determining the extent to which the **Perspective-Taking** disclosures directly pertain to participants’ selves.

The claims on disclosure are with respect to multiple dimensions: quantity via word counts and abstractions, and depth via information, thoughts, and overall. However, the disclosure of *feelings* remains an area for deeper investigation. Within the present study, few disclosures pertained to participant emotions or feelings, regardless of condition. Roughly 96% of the responses across both conditions were assigned (depth = 1) no disclosure of feelings. Since both conditions disclosed little emotional content, this may seemingly be explained by the fact that each of the nine disclosure items directly requested disclosure of information (e.g., experiences, background) or thoughts (e.g., opinions, goals, plans). While our reflective conversation did assess participants’ feelings and emotions, such sentiments were primarily captured in the closed-ended items, which could not be included in the analysis of the disclosure items. As a result, the responses to the nine disclosure items almost exclusively pertained to participants’ direct histories, experiences, thoughts, and opinions. However, the lack of emotional expression may also reflect a broader limitation of perspective-taking itself, which may enhance depth and thought but not necessarily encourage the disclosure of affective content. This possibility invites further investigation into whether perspective-taking facilitates cognitive but not emotional forms of disclosure.

### 4.2 AI chatbots to promote mental wellbeing

In light of findings on readiness, we discuss how perspective-taking led to such effects, implications for wellbeing chatbot interactions, and design considerations based on user attitudes.

While both conditions experienced significantly improved readiness outcomes, our findings suggest that the degree of improvement between conditions was not significantly different (see Figure 3). Though hypotheses were deferred in favor of **Perspective-Taking**, it would also reason that speaking from one’s own perspective (**Control**) should naturally afford disclosures more pertinent to the self, as well as more self-tailored expressions of emotion from the chatbot. We provide several possible explanations of how **Perspective-Taking** disclosures may have led to seemingly comparable user outcomes. First, the improved readiness observed in **Perspective-Taking** likely reflects previously discussed mechanisms of activated motivations to change and blurred boundaries of helping the self or an other (66, 76, 77, 81, 82). And while potential self-other overlap effects may also account for such improvements, another plausible possible factor is the therapeutic effect of written emotional experiences, which has been shown to enhance wellbeing (143, 144). Written emotional disclosures may cover topics such as emotional experiences, future aspirations, or past successes, akin to topics in our reflective conversation. Prior work has found that writing forms of emotional disclosures can help facilitate relief for anxiety and depressive symptoms (145–147), with improvements in self-esteem (148) and even physical health symptoms (149–151). Interestingly, some studies have investigated the effects of written emotional disclosures from non-self perspectives. Greenberg et al. found that writing emotional disclosures from an unexperienced, imagined perspective led to improvements in health symptoms and lower immediate reports of depression, fatigue, and avoidance (152). King et al. also found that writing from a distance perspective in the form of a hypothetical (ideal) self could elicit similar health effects in comparison to writing about self-experiences (146). Our findings echo prior work illustrating that writing emotional disclosures, regardless of perspective, may produce positive effects on the self. Finally, it is also worth briefly mentioning the potential role of observed enhancements in disclosures in **Perspective-Taking**. The findings suggest that **Perspective-Taking** participants wrote more quantity with greater depth than the **Control**, which may have further contributed to overall improvements and lack of significant differences between conditions. No formal analysis was able to be conducted on the relationship between disclosures and readiness, but such investigations could further elucidate how chatbots can promote self-outcomes.

Based on our findings and discussion, it would appear that direct self-disclosures may not be a strict requirement to promote user outcomes with chatbots. This may have numerous implications for pathways to promote interactions with emotional intelligence AI. In chatbot conversations addressing highly stigmatized topics (e.g., severe health issues, sexual health, or mental health), users may limit their disclosures due to shame or fear of judgment (29, 30, 153). If our findings hold, a distanced perspective may be able to be leveraged to overcome such stigmas and draw deeper disclosures for self-benefit. A similar domain that applies such techniques is within therapeutic role-plays, which has demonstrated that *imaginative* scenarios can be employed for self-understanding, improvement, and behavior (154–156). The present study also supports preliminary findings suggesting that such engagements may be suitable for human-computer simulations (157). Another implication of our work is towards the ethical, safe usage of AI chatbots, especially with regard to user data privacy and security. Though chatbots have been shown to be a promising opportunity for health outcomes (3, 5, 6), concerns arise in the employment of *generative AI* for wellbeing. Generative AIs can run the risk of memorizing or reproducing data, which poses further considerations in digital health conversations that pertain to protected or sensitive health information (158). Generative AI also faces broad technology risks associated with compromised data and leaks (159). While cybersecurity safeguards and processing data in a de-identified state can provide a layer of security, even de-identified information can potentially be re-identified with real persons (158). Perspective-taking may offer a potential mitigation strategy for these risks. By encouraging distanced disclosure, users may still benefit from reflective engagement without exposing identifiable or sensitive information. Such efforts align with recent ethical recommendations that emphasize a need to minimize data exposure in AI-mediated mental health contexts (160, 161). Given skepticism findings suggesting that the present intervention was perceived as less relevant to perspective-takers, distanced perspectives may be able to help buffer against negative effects that arise from AI hallucinations, since inaccurate or misleading responses may not necessarily interpreted as personally relevant or diagnostic (162). In this way, our study contributes to ongoing discussions about how to design AI systems that are both effective and ethically responsible in sensitive domains.

The promising findings on disclosure and readiness should be interpreted in light of the more complex pattern observed in attitudes. Perspective-taking may be able to promote disclosure behaviors, but it may come at a cost of perceived personal applicability of the chatbot’s support. Worth noting is that the skepticism measure was employed to gauge perceptions of the intervention’s ability to provide *effective, personal* support, and the resulting attitudes would be consistent with expectations that **Perspective-Taking** would include less self-relevant disclosures and/or support compared to **Control**. The findings of **H5** and **H6** are also aligned with established literature on self-distancing and construal-level theory that illustrate that distance reduces egocentric experiences with stimuli and leads to less self-relevant appraisals (44, 163). In other words, when individuals adopt another person’s perspective, they may feel less directly connected to the experience and perceive it as less personally relevant or useful, even if it encourages thoughtful reflection. Given the role that attitudes may play in one’s decision to engage with such digital interventions (164), the divergence in attitudes warrants additional considerations. The confounding effect raises practical concerns for the design of supportive AI systems that leverage psychological distance. A perceived lack of belief that the AI can support the user may lead to diminished future engagements (165), even if the intervention effectively prompts deeper disclosure in the present. Users may also be resistant to advice from the AI chatbot, despite its potential effectiveness due to distancing effects and relevance (166). If perspective-taking prompts deeper reflection but undermines one’s attitudes towards using such systems, its standalone use may be insufficient. Perspective-taking and distancing theories could enhance wellbeing chatbot engagements, but may need to be complemented with strategies that restore personal resonance to foster congruently positive attitudes. Future work could investigate practices in therapeutic contexts where distancing is spontaneous rather than longitudinal (163) or patients switch between immersed and distanced perspectives (167).

### 4.3 Limitations and future work

There are limitations to this study that help contextualize its findings and identify avenues for future research. The focus of this work was on driving disclosure and wellbeing among university-attending populations with AI; as such, recruitments were made through a University of Florida student research platform. The resulting population consisted primarily of STEM students, which limits the generalizability of the results to broader student populations. STEM students may have more familiarity with AI compared to general populations and differing mental health concerns that may alter their usage of such systems (168). Furthermore, the present work involved a large qualitative corpus of 1,479 codes for participant disclosures, but successful capture of the lost data may have allowed greater ability to analyze relationships between disclosures and outcomes. The lack of emotional disclosures from this structured, reflective conversation also limits the ability to understand how such methods can elicit affective engagement. Understanding these relationships could further clarify the role of disclosure as a mediating factor in AI chatbot engagements. A few study design limitations are mentioned for future work to help validate research with perspective-taking and emotionally intelligence AI chatbots. While the present perspective-taking intervention appeared effective, the absence of a placebo condition limits our ability to isolate the effects of intervention content from outstanding engagement effects. The results of this work are interpretable within single-session mental wellbeing conversations. As a result, the present outcomes are confined to immediate effects on disclosure and readiness to address mental wellbeing, but longitudinal effects on disclosure or actual changes in healthy behavior remain an unexplored area for future work. The intervention also relied on a carefully structured set of tasks to elicit the perspective within an asynchronous environment. Wellbeing interventions may struggle to incorporate such specific tasks within their contexts. Although prior research indicates that perspective-taking can happen more spontaneously (94, 95), future integrations may need to consider alternative methodologies to integrate distancing practically, especially with respect to the attitudinal findings on skepticism. Several related areas for investigation outside of the current scope of work include: perceptions of the chatbot’s expression of emotion, participants’ attitudes toward their designated, or self–other overlap. Future work should continue to research the noted limitations, as well as opportunities to employ potential implications of the present work.

## 5 Conclusion

AI chatbots continue to act as a medium towards reducing barriers for mental support, especially when supplemented with emotional intelligence. However, the capabilities of such chatbots and their resulting outcomes can be limited without meaningful engagements and disclosures from users. A conversation with little-to-no depth may only elicit a surface-level understanding of the wellbeing concerns and needs of an individual. Similar to support from a counselor, a friend, or loved one, a chatbot’s capability to appropriately assist and empathize may increase when provided with a greater quantity and depth of context. The findings of this study illustrate that perspective-taking may be able to enhance disclosure to AI chatbots for wellbeing. Specifically, our results suggest that perspective-taking led participants to share significantly greater disclosure in forms of word quantity and depth across multiple categories, with limited evidence of abstractions beyond what was seen in our control. Furthermore, the AI chatbot intervention seemingly helped all participants improve their readiness and intentions to address mental wellbeing, and perspective-taking did not seemingly diminish the gain in participants’ improvements. In light of prior literature, our findings may suggest that meaningful disclosure to chatbots to improve mental wellbeing readiness may not necessarily require direct self-disclosures. In doing so, we describe implications for how perspective-taking and distancing theories may further enhance disclosure to chatbots in sensitive contexts or in pursuit of minimizing the disclosure of sensitive self-information. Future work should continue to investigate how greater disclosure can be evoked to meaningfully foster user outcomes with emotionally intelligent AI chatbots based on the limitations and emergent gaps identified in the present study.

## Data Availability

The raw data supporting the conclusions of this article will be made available by the authors, without undue reservation.

## Footnotes

^1^ https://github.com/met4citizen/TalkingHead

^2^ https://www.ims.uni-stuttgart.de/en/research/resources/tools/treetagger/
