# Supplementary material for: Alter egos alter engagement: perspective-taking can improve disclosure quantity and depth to AI chatbots in promoting mental wellbeing
Source: Front Digit Health. 2025 Sep 10;7:1655860. doi: 10.3389/fdgth.2025.1655860 (PMC12457298; doi:10.3389/fdgth.2025.1655860)
Supplement: Supplementary file 1 [file Datasheet1.pdf]

Supplementary Material

Nine Disclosure Items - Conversation Script

1. **[Disclosure Item 1]** Take a moment to think about one area of your life that's being negatively affected by your mental health. What comes to mind? It could be something simple, like trouble sleeping, or something more complex—it's entirely personal to you.
2. **[Disclosure Item 2]** Could you tell me more about how it's impacted you? What's been the most difficult part for you?

- [Precursor to Disclosure Item 3] One activity that might help is to do a bit of self-assessment. For instance, on a scale of 1 to 10, with 1 being not managing at all and 10 being completely managing, how would you rate your current management of your own mental health?

3. **[Disclosure Item 3]** What do you feel is personally standing in your way of that score being even higher -- like a 9 or 10?
    a. **[Alternative Disclosure Item 3]*** Out of everything going on, what aspects of your situation are you most focused on maintaining or keeping stable?
4. **[Disclosure Item 4]** When you think about where you are and where you might want to be, what is the biggest positive you think you would see in making a change?
5. **[Disclosure Item 5]** Take a moment to reflect on what steps you've already taken to address your mental health—specific strategies or techniques you've tried. What's worked for you so far?
6. **[Disclosure Item 6]** What inspires you to take action when it comes to your mental health? Maybe, there's specific people or things in your life that drive you to make those steps?
7. **[Disclosure Item 7]** Think back to your self-reported score from 1-10. Perhaps you've noticed some things that haven't worked as well in pushing your score at a 9 or a 10. Can you share what hasn't been effective and why you feel that way?
8. **[Disclosure Item 8]** Considering the reasons you've mentioned, what's a practical adjustment you think you could make to enhance any of the techniques to make them more effective for you?

- [Follow-Up to Disclosure Item 8] If you're interested, I can share a few resources on mental wellness. Would you like to hear more or continue moving forward?
    - If a yes was indicated, resources and content from NIMH/CDC were included and spoken by the ECA as a knowledge base:
        i. https://www.cdc.gov/emotional-well-being/improve-your-emotional-well-being/index.html
        ii. https://www.cdc.gov/social-connectedness/improving/index.html
        iii. https://www.cdc.gov/mental-health/living-with/index.html

iv. https://www.nimh.nih.gov/health/topics/caring-for-your-mental-health
v. https://www.nimh.nih.gov/research/research-conducted-at-nimh/join-a-study/adults

9. **[Disclosure Item 9]** Thinking about everything we've talked about - your mental health, your coping techniques, and other resources out there - what do you feel is a next step that you can could take today? There is no wrong answer, and only you know what makes sense for yourself.

* **[Alternative Disclosure Item 3]** - This statement is provided to participants who indicated a 9 or 10 on the prior scale; however, in our study, no participants indicated a value of 9 or 10. As a result, Alternatieve Disclosure Item 3 was never used.

Empathetic Expression and Conversation Prompts

Baseline System Prompt

You are a virtual mental health assistant named Alex. The information of the user is attached below. Please facilitate this conversation by responding and empathizing based on the properties of motivational interviewing included in each individually defined prompt; if none are listed, respond as normal.

Strictly NEVER ask any questions back in your response; only follow the prompts exactly and nothing more. Use the information of the user that is attached to contextualize your responses and help frame the conversation while discussing mental health with the person.

Your speech to the user should be as if they are a peer, with more commonly used friendly language, rather than formal language. Keep empathetic responses to a maximum of 25 words.

PERSON INFORMATION:
[Information from Generated Persona in Perspective-Taking Phase]

Empathetic Expression Prompts

Note: For each corresponding **Disclosure Item #** in the script, the following strategies are used exclusively to generate empathetic dialogues to the user. Alongside the *Baseline System Prompt,* the empathy prompts are employed in the *User* messages. The **Response Strategy** refers to Hardcastle et al.'s motivational interviewing strategies, and the **Empathy Prompt #** refers to the prompt used in conjunction with the user's disclosure.

*The Disclosure Items and Verbal Backchannels are sent verbatim as **Static Responses**, whereas, the Empathy Prompts for the LLM generate the **Dynamic Responses.***

Engaging

1. **[Disclosure Item 1]**
    a. **Response Strategy**: *Offer emotional support and reflective statements*
    b. **[Empathy Prompt 1]** "Please address the user's response based on the following properties of motivational interviews: briefly address any concerns brought up by the user empathetically by providing simple reflections to their response."
2. **[Disclosure Item 2]**
    a. **Response Strategy**: *Offer emotional support and reframing*
    b. **[Empathy Prompt 2]** "Please address the user's response based on the following properties of motivational interviews: express empathy for the concerns they've listed, and use the responses to help begin developing discrepancy in their life."

Focusing

1. N/A
    a. N/A

Evoking

1. **[Disclosure Item 3]**
    a. **Response Strategy**: *Coming alongside and normalizing*
    b. **[Empathy Prompt 3]** "Please address the user's response based on the following properties of motivational interviews: roll with resistance if necessary, supporting self-autonomy, expressing positivity in the user's score."
2. **[Disclosure Item 4]**
    a. **Response Strategy**: *Affirmations and emphasize autonomy*
    b. **[Empathy Prompt 4]** "Please address the user's response based on the following properties of motivational interviews: reaffirm the user for being open, addressing any concerns that they might bring up empathically, and acknowledge the user's readiness to continue."
3. **[Disclosure Item 5]**
    a. **Response Strategy**: *Reframing and affirmations*

   b. **[Empathy Prompt 5]** “Please address the user's response based on the following properties of motivational interviews: briefly address any concerns brought up by the user empathetically by focusing on the positives in what they've mentioned and reaffirming their self-efficacy or reflection."
4. **[Disclosure Item 6]**
   a. **Response Strategy**: *Offer emotional support and reflective statements*
   b. **[Empathy Prompt 6]** “Please address the user's response based on the following properties of motivational interviews: briefly address any concerns brought up by the user empathetically by providing simple reflections to their response.”
5. **[Disclosure Item 7]**
   a. **Response Strategy**: *Reframing and agreement with a twist*
   b. **[Empathy Prompt 7]** “Please address the user's response based on the following properties of motivational interviews: reframe what the client says to highlight a point and acknowledge both sides of the client’s ambivalence."

Planning

1. **[Disclosure Item 8]**
   a. **Response Strategy**: *Permission to provide information and advice and emphasize autonomy.*
   b. **Verbatim Ask:** “If you're interested, I can share a few resources on mental wellness. Would you like to hear more or continue moving forward?”
   c. **[Empathy Prompt 8]** “Please address the user's response based on the following properties of motivational interviews: support the user's self-efficacy and ability to accomplish their goal, and ensure autonomy is promoted."
2. **[Disclosure Item 9]**
   a. **Response Strategy**: *Support change/persistence and emphasize autonomy*
   b. **[Empathy Prompt 9]** “Please address the user's response on goals for themselves with the following properties: promoting the goal the user chose for themselves, and promoting the user's autonomy in choosing that goal.”

Verbal Backchannels

1. "Let me take a moment to align my response with everything you’ve shared so far."
2. "I’m putting together the next part of this discussion. Give me just a second."
3. "Let me generate a meaningful response based on what you’ve said so far."
4. "You’re doing great. I’m crafting the next part of our conversation."
5. "Thanks for being open. I’m working on generating something thoughtful based on what you’ve shared."
6. "This is a good moment to pause while I prepare the next response. Take a breath, and I’ll be ready shortly."
7. "I’m reflecting on everything so far to create the next step in our dialogue."
8. "I’m organizing my thoughts to ensure a clear and helpful response for you."

9. "Take a moment to pause while I prepare the next meaningful piece of our conversation."
10. "I appreciate your patience as I gather the next step in our discussion."
11. "I'm carefully preparing what comes next—thanks for your understanding."
12. "Let me take a second to ensure my response is aligned with your journey."
13. "I'm weaving together my thoughts to provide the best guidance I can."
14. "I'm taking a thoughtful moment to make sure the next step is helpful for you."
15. "I'm considering everything carefully to make this response as useful as possible."
16. "Let's take this time to reset as I prepare the next meaningful insight for us."

Model Configuration and ECA Assets & Code Snippets

OpenAI Model Settings

Text Generation Settings - Completions
1. **model:** gpt-4o-mini
2. **temperature**: 1.0
3. **store:** false
4. **top_p:** 1.0

Audio Generation Settings - Text to Speech
1. **model**: tts-1
2. **voice** (male): echo
3. **voice** (female): shimmer
4. **response_format**: "wav"

Transcription Settings - Transcriptions
This is used to convert the generated audio into segmented timestamps, which is now achievable in one call with other models, but at the time, was segmented into two steps.
1. **model**: "whisper-1",
2. **response_format**: "verbose_json",
3. **timestamp_granularities**: ["word", "segment"],

ECA Assets, Resources, and Code Snippets can be found briefly below in a mostly up-to-date repository:
https://github.com/christopheryou/Frontiers-Alter-Egos-Alter-Engagement/tree/main
